# N-acetylcysteine as a potential treatment for novel coronavirus disease 2019

**DOI:** 10.2217/fmb-2020-0074

**Published:** 2020-07-14

**Authors:** Rangel-Méndez Jorge-Aarón, Moo-Puc Rosa-Ester

**Affiliations:** ^1^Unidad de Investigación Biomédica, Unidad Médica de Alta Especialidad, Hospital de Especialidades del Centro Médico Nacional ‘Ignacio García Téllez’, Instituto Mexicano del Seguro Social, Calle 41 No. 439, Col. Industrial, 97150 Mérida, Yucatán, México

**Keywords:** COVID-19, N-acetylcysteine, SARS-CoV-2

The novel coronavirus (CoV) disease 2019 (COVID-19), which first appeared in Wuhan, China, in December 2019, spreads efficiently from person to person. After it had reached over 100 countries, on 11 March 2020 the WHO declared it a pandemic [1]. COVID-19 is caused by SARS-CoV-2, and by 9 June 2020 had been responsible for 7,039,918 confirmed cases and 404,396 deaths worldwide [2]. At the time of writing, the five countries with the highest number of cases are the USA (1,933,560 cases), Brazil (691,758 cases), the Russian Federation (485,253 cases), the UK (287,403 cases) and India (266,598 cases) [2].

The scientific community’s rapid response has allowed description of the complete SARS-CoV-2 genome, which is currently available on bioinformatics platforms. Analysis of the genome has found an 88% identity with two bat-derived SARS-like CoVs, bat-SL-CoVZC45 and bat-SL-CoVZXC21, both collected in 2018 in Zhoushan, Eastern China; it also has approximately 79% identity with 2002 SARS-CoV [3]. It is no surprise therefore that SARS-CoV-2 shares host cell infection mechanisms with SARS-CoV. Angiotensin-converting enzyme 2 (ACE2) has been shown to be the receptor in which the SARS-CoV-2 spike (S) glycoprotein allows membrane fusion and internalization [4]. The SARS-CoV-2 S glycoprotein bonds to ACE2 resulting in reduced expression of the enzyme; this generates angiotensin II accumulation generated by ACE. The depleted ACE2 is unable to convert angiotensin I into the vasodilator heptapeptide angiotensin 1–7, thus generating pulmonary injury; also, angiotensin II type-1 receptor overstimulation results in increased lung vascularity which contributes to the overall pathology. Human ACE2 and the SARS-CoV-2 S glycoprotein have consequently been identified as the therapeutic targets for development of new treatments such as antivirals and monoclonal antibodies, or for identification of existing drugs capable of blocking interaction between the virus and the host cell.

The SARS-CoV-2 S glycoprotein consists of two subunits, S1, which facilitates viral bond to the host cell, and S2, which assists viral membrane fusion [5]. The fusion process depends on S glycoprotein cleavage at the S1/S2 multibasic site, mainly by the human protease furin [6]. *In vitro* results demonstrate the essential role of this cleavage site to promote viral entrance into lung cells [6]. Thus, direct inhibition of furin or disruption of the interactions between the S1/S2 complex and this protease are potential therapeutic approaches.

We propose N-acetylcysteine (NAC) as a potential treatment, preventive and/or adjuvant against SARS-CoV-2. It has two principal activities: NAC exhibits a mucolytic effect due to its free sulfhydryl group which reduces disulfide bonds in the cross-linked mucus glycoproteins matrix, thereby lowering mucus viscosity [7]; and NAC is a potent antioxidant with a direct effect on certain oxidant species, an indirect effect because it acts as a precursor to cysteine (required for glutathione synthesis), and the ability to restore thiol pools which in turn regulate redox state [7].

Considering these properties, we hypothesize that NAC could negatively affect SARS-CoV-2 activity for the following reasons:

- The E protein of SARS-CoV (genetically related to SARS-CoV-2) consists of 76–109 amino acids, ranging in size from 8.4 to 12 kDa. Its primary and secondary structures have a short, hydrophilic amine terminus group of 7–12 amino acids followed by a hydrophobic 25 amino acid transmembrane domain which ends in a hydrophilic carboxyl group terminus [8]. The SARS-CoV-2 E protein includes a triple cysteine motif (NH_2_- … L-Cys-A-Y-Cys-Cys-N … -COOH) after the transmembrane domain which interacts with a similar motif from S protein terminal C- (NH_2_- … S-Cys-G-S-Cys-Cys-K … -COOH) [8]. Both motifs interact through disulfide bonds [8], and NAC may cleave them. This would decrease SARS-CoV-2 infectivity.
- *In vitro* studies have shown NAC to decrease angiotensin II bonds to angiotensin II type 1 receptor in a dose-dependent manner [9]. In the COVID-19 context, NAC could block excessive production of angiotensin II, which cannot be cleaved to angiotensin 1–7 by ACE2. This may decrease pulmonary disease severity.
- *In vitro* and clinical studies have shown NAC to block ACE. In one experiment isosorbide dinitrate (vasodilator activity) was administered to six male participants for 48 h, but at 24 h NAC was added (2 g intravenously [iv.] followed by 5 mg/kg/h). Angiotensin II plasma concentrations increased during the first 24 h of isosorbide dinitrate administration but just 2 h after NAC initiation they had decreased from 28 ± 4 to 14 ± 2 ng/l (p < 0.05) [10]. This suggests that, by blocking ACE, NAC may provide protection from the deleterious effects of angiotensin II, a potentially useful activity in a SARS-CoV-2 infection scenario.
- The oxidative stress environment created by cytokine storm syndrome and production of reactive oxygen species (ROS) may be attenuated by NAC’s antioxidant effect [11]. Also, the SARS-CoV-2 immunopathology may be similar to that of SARS-CoV, which generates an immune response involving diverse pro-inflammatory cytokines (IL-1, IL-2, IL-4, TNF and IFNs). The IFNs are classified in type-I (IFN-α and β), -II (IFN-γ) and -III. Type-I IFNs are suppressed during SARS-CoV infection due to impairment of signal transducer and activator of transcription 1, which ultimately antagonizes IFN. This complex mechanism may also generate delayed IFN response due to cytokine storm syndrome during SARS-CoV-2 infection, possibly explaining COVID-19 pathology. NAC may amplify the signaling functions of toll-like receptor 7 and mitochondrial antiviral signaling protein in restoring type-I IFN production during SARS-CoV-2 infection [11].
- NAC has been shown to restore platelet GSH reserves (in a murine model) which in turn can prevent protein glycosylation by methylglyoxal, a pathologic mechanism in diabetic patients [12]. The SARS-CoV-2 S glycoprotein differs from that of SARS-CoV in that it gains new glycosylation sites (NGTK, NFTI, NLTT and NTSN), allowing SARS-CoV-2 to enter the host cell [5]. Administration of NAC could prevent additional glycosylation events in SARS-CoV-2, thus inhibiting its infectivity and any associated pathologies.
- In a recent study the NF-κB was described as a mediator of SARS-CoV-2 pulmonary pathology since it triggers the production of numerous pro-inflammatory cytokines. This process generates macrophage and neutrophil infiltration, both of which cause irreparable damage to pulmonary epithelium cells. NAC was shown to inhibit NF-κB activation in an *in vitro* influenza (A and B) model [13]; the proposed mechanism is restauration of thiol pools which may allow ROS scavenging. This is relevant because recent clinical experience has shown that severity of COVID-19 clinical manifestations might be associated with decreased GSH levels and the consequent increased ROS production. Severe COVID-19 cases would therefore probably manifest lower GSH levels, higher ROS levels and greater redox status (ROS/GSH ratio) than milder cases [14].
- In the context of influenza virus infection, NAC administration (100 mg/kg continuous iv. infusion daily for 3 days) was reported to promote clinical improvement in a woman with H1N1 influenza pneumonia; oseltamivir was also employed during treatment [15]. However, other studies have found no beneficial *in vitro* or *vivo* effects with NAC administration [16]. NAC (600 mg twice daily) has also been reported to attenuate influenza symptoms in patients ≥65-years old with chronic-degenerative diseases [17].

Given this pandemic’s immense health risk, several drugs have been employed with and without clinical evidence for the treatment of COVID-19, NAC among them [18]. Administration of NAC (oral, iv. or inhaled) as an adjuvant treatment in patients with mild–severe COVID-19 symptoms is worth considering as a cost–effective clinical strategy. Currently, there are some clinical trials assessing the potential use of NAC against COVID-19; for example, the ‘Efficacy and Safety of Nebulized Heparin-N-acetylcysteine in COVID-19 Patients by Evaluation of Pulmonary Function Improvement (HOPE)’ clinical trial is aimed at determining the efficacy of nebulized NAC and heparin in ventilated COVID-19 patients [19]. The aim is to increase ventilator-free days in hospitalized patients with moderate–severe COVID-19 symptoms. Another recent study is ‘A Study of N-acetylcysteine in Patients With COVID-19 Infection’, a clinical trial aimed at quantifying: the number of patients successfully extubated and/or transferred from critical care unit due to clinical improvement; and the number of patients discharged due to clinical improvement. Patients are receiving NAC iv. 6 g/day in addition to other treatments prescribed for COVID-19 [20].

Oral administration of NAC (600 mg/day) could function as a preventive measure, particularly in those repeatedly exposed to possible SARS-CoV-2 carriers (e.g., health workers). This application could be a particularly urgent approach since, despite the use of personal protective equipment, healthcare workers in the USA, Italy, China, Mexico, etc., have become infected while caring for hospitalized patient. Other workers who, due to their job requirements, cannot work at home and/or ensure self-isolation might also benefit from preventive use of NAC administration. If deemed effective, this latter use could potentially help to flatten the exponential contagion curve in several countries. More clinical trials would clearly be needed to validate this application.

Basic laboratory and clinical studies are required to confirm possible use of NAC as an element in combating the disease caused by SARS-CoV-2. This would need to be one of myriad efforts to identify additional treatments (novel or not) aimed at halting the current COVID-19 pandemic, or at the very least slowing person-to-person contagion.

## References

[1] World Health Organization. WHO Director-General’s opening remarks at the media briefing on COVID-19 (2020). https://www.who.int/dg/speeches/detail/who-director-general-s-opening-remarks-at-the-media-briefing-on-covid-19---11-march-2020?fbclid=IwAR25Aw5IrZOqg7znGfG3OdUKBN2Hw8WL4kHHVTPEv8EdJOqg3JD-vLz1HQY

[2] World Health Organization. Coronavirus disease (COVID-19) situation dashboard (2020). https://covid19.who.int/

[3] Lu R, Zhao X, Li J Genomic characterisation and epidemiology of 2019 novel coronavirus: implications for virus origins and receptor binding. Lancet 395(10224), 565–574 (2020). 32007145 10.1016/S0140-6736(20)30251-8 PMC7159086

[4] Wan Y, Shang J, Graham R, Baric RS, Li F Receptor recognition by novel coronavirus from Wuhan: an analysis based on decade-long structural studies of SARS coronavirus. J. Virol. 94(7), e00127–20 (2020). 31996437 10.1128/JVI.00127-20 PMC7081895

[5] Kumar S, Maurya VK, Prasad AK, Bhatt MLB, Saxena SK Structural, glycosylation and antigenic variation between 2019 novel coronavirus (2019-nCoV) and SARS coronavirus (SARS-CoV). Virus Disease 31(1), 1–9 (2020). 32206694 10.1007/s13337-020-00571-5 PMC7085496

[6] Hoffmann M, Kleine-Weber H, Pöhlmann S A multibasic cleavage site in the spike protein of SARS-CoV-2 is essential for infection of human lung cells. Mol. Cell. 78(4), 779–784.e5 (2020). 32362314 10.1016/j.molcel.2020.04.022 PMC7194065

[7] Aldini G, Altomare A, Baron G N-acetylcysteine as an antioxidant and disulphide breaking agent: the reasons why. Free Radic. Res. 52(7), 751–762 (2018). 29742938 10.1080/10715762.2018.1468564

[8] Schoeman D, Fielding BC Coronavirus envelope protein: current knowledge. Virol. J. 16(1), 1–22 (2019). 31133031 10.1186/s12985-019-1182-0 PMC6537279

[9] Ullian ME, Gelasco AK, Fitzgibbon WR, Beck CN, Morinelli TA N-acetylcysteine decreases angiotensin II receptor binding in vascular smooth muscle cells. J. Am. Soc. Nephrol. 16(8), 2346–2353 (2005). 15944340 10.1681/ASN.2004060458

[10] Boesgaard S, Aldershvile J, Poulsen HE, Christensen S, Dige-Petersen H, Giese J N-acetylcysteine inhibits angiotensin converting enzyme *in vivo*. J. Pharmacol. Exp. Ther. 265(3), 1239–1244 (1993). 8389858

[11] McCarty MF, DiNicolantonio JJ Nutraceuticals have potential for boosting the type 1 interferon response to RNA viruses including influenza and coronavirus. Prog. Cardiovasc. Dis. (2020) (Epub ahead of print). 10.1016/j.pcad.2020.02.007 PMC7130854 32061635

[12] Wang B, Yee Aw T, Stokes KY N-acetylcysteine attenuates systemic platelet activation and cerebral vessel thrombosis in diabetes. Redox Biol. 14, 218–228 (2018). 28961512 10.1016/j.redox.2017.09.005 PMC5619994

[13] Mata M, Morcillo E, Gimeno C, Cortijo J N-acetyl-L-cysteine (NAC) inhibit mucin synthesis and pro-inflammatory mediators in alveolar type II epithelial cells infected with influenza virus A and B and with respiratory syncytial virus (RSV). Biochem. Pharmacol. 82(5), 548–555 (2011). 21635874 10.1016/j.bcp.2011.05.014

[14] Polonikov A Endogenous deficiency of glutathione as the most likely cause of serious manifestations and death in COVID-19 patients. ACS Infect. Dis. (2020) (Epub ahead of print). 10.1021/acsinfecdis.0c00288 32463221

[15] Lai KY, Ng WY, Chan PKO, Wong KF, Cheng F High-dose N-acetylcysteine therapy for novel H1N1 influenza pneumonia. Ann. Intern. Med. 152(10), 687–688 (2010). 20479037 10.7326/0003-4819-152-10-201005180-00017

[16] Garigliany MMO, Desmecht DJ N-acetylcysteine lacks universal inhibitory activity against influenza A viruses. J. Negat. Results Biomed. 10(1), 5 (2011).21554703

[17] De Flora S, Grassi C, Carati L Attenuation of influenza-like symptomatology and improvement of cell-mediated immunity with long-term N-acetylcysteine treatment. Eur. Respir. J. 10(7), 1535–1541 (1997). 9230243 10.1183/09031936.97.10071535

[18] Fajgenbaum DC, Khor JS, Gorzewski A Treatments administered to the first 9152 reported cases of COVID-19: a systematic review. Infect. Dis. Ther. (2020) (Epub ahead of print). 10.1007/s40121-020-00303-8 PMC7251321 32462545

[19] Quay S COVID-19 treatment HOPE protocol (2020). https://drquay.com/covid-19-doctor/

[20] Memorial Sloan Kettering Cancer Center. A study of N-acetylcysteine in patients with COVID-19 infection (2020). https://clinicaltrials.gov/ct2/show/NCT04374461

